# Engineering an Interdisciplinary Connection: Bridging Gaps between Chemical, Electrical, and Environmental Engineers

**DOI:** 10.1016/j.isci.2020.101337

**Published:** 2020-07-20

**Authors:** Yury Dvorkin, Miguel A. Modestino, Andrea I. Silverman

**Figure:**
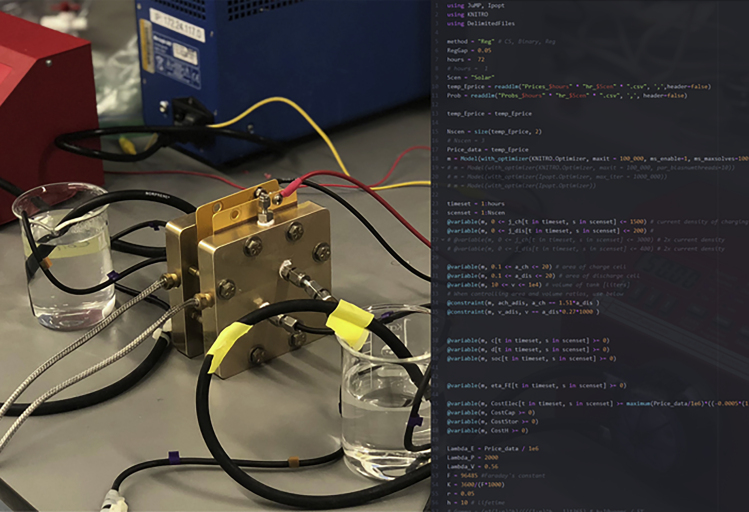
In this photo, the experimental dual-use energy storage and hydrogen generation cell built by Modestino's lab (with expertise in chemical engineering) is shown with the optimization code built by Dvorkin's lab (with expertise in electrical engineering), highlighting the very different media that each discipline focuses on in the collaboration.

## Small Summary of Your Research Collaboration

Society depends on access to water, electricity, materials, and fuels, and these infrastructure networks are uniquely interlinked. Given this interconnection, it is important that experts in each area, often coming from very different disciplinary backgrounds, consider possible interdependencies. Three assistant professors at New York University's Tandon School of Engineering (NYU Tandon)—environmental engineer Andrea Silverman, electrical engineer Yury Dvorkin, and chemical engineer Miguel Modestino—are combining their distinct expertise to tackle complex societal problems at the intersection of their fields in a way that is often precluded by unique language and conventions used in each of their disciplines. Below, they share their story about building their collaborations, finding a common language, and making strides as early career pioneers of interdisciplinary research.

Prof. Yury Dvorkin, Prof. Miguel Modestino, and Prof. Andrea Silverman, all assistant professors at NYU Tandon, have undertaken interdisciplinary research and collaborations early in their careers to engage students, seize funding opportunities, and try to tackle real-world problems facing engineers.

## Proximity: Who were the players in this project, and how did you bring everyone together?

We all started as assistant professors at NYU Tandon within a year of each other. In starting our careers as faculty members, we were individually interested in including interdisciplinary projects in our research programs, given that distinct, yet complementary, skillsets are needed to tackle complex societal challenges. NYU Tandon emphasizes and prioritizes collaborative work and promotes interaction between faculty members across its departments. It was during school-sponsored junior faculty gatherings and small group lunches that we first met and built connections. It was at a multidisciplinary research seminar highlighting new faculty where we learned more deeply about each other's research programs. Additionally, NYU Tandon has a motto that we do *Engineering in Service to Society*. While our collaborations were meaningful to us personally, they were also supported by our institution.

## Language: Did you encounter any challenges or any benefits of working with people from different backgrounds and expertise? How did you bridge the language gap among different disciplines?

A large language barrier exists between chemical or environmental engineers, who describe transformation of matter in the form of chemical reactions, and electrical engineers, who use complex mathematical formulations to describe the interaction of the electricity network with its physical components.

**Figure:**
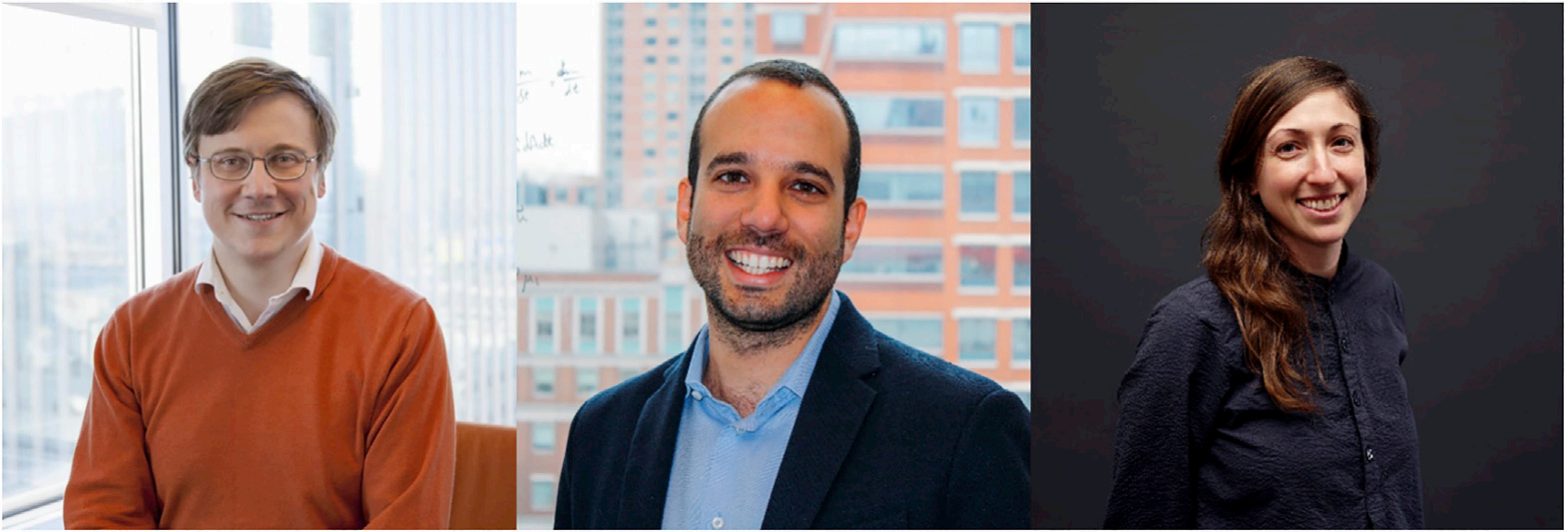

This gap meant that significant effort and time was needed to learn each other's language, which often involved writing and editing proposals on a shared document, brainstorming ideas on the board, and going through multiple iterations to establish a common language between the fields. For example, we had to work together to translate the behavior of electrosynthetic reactors into mathematical formulations that could be embedded into power grid optimization models. Ironically, the developed reactor formulations belong to a different class of mathematical optimization models than grid models and, therefore, the team needed to develop a surrogate model that would allow for jointly optimizing the storage reactors and grid. As a result, the surrogate models were trained by experimental data that correlated the performance of the electrochemical devices with the inputs that they receive from the power grids. These models then could be used to identify the optimal scheduling of the operation of the reactors to maximize their profitability when integrated with a real power grid.

## Research Methods: Did this project require tailoring your research methods to adjust to working interdisciplinarily?

During the collaboration, although vocabulary was a hurdle to overcome between chemical and electrical engineering, we found it interesting that chemical and environmental engineering use a similar vocabulary to discuss fundamental principles shared by the two fields, such as mass and energy balances, reaction kinetics, and reactor designs. Experimentally, both chemical and environmental engineering can involve material synthesis, degradation, or transformation. However, we found that the target compounds and solutions that both fields work with, and the way they are described, can differ dramatically between the two fields.

For example, chemical engineers tend to apply the fundamental principles described above to applications where experimental conditions are controlled and well characterized (i.e., known concentrations of pure reagents). Alternatively, environmental engineers apply the same principles to more complex, heterogeneous matrices, such as domestic wastewater, sewage sludge, and environmental water samples, including water and sediment from lakes, rivers, wetlands, and subsurface and marine environments. These uncontrolled solutions can vary greatly between locations and over time, which can present additional challenges in designing reactors and evaluating their performance. Furthermore, environmentally relevant abundances of target compounds can be much lower than what are typically found in chemical processes but can still present risks to environmental quality or public health.

**Figure:**
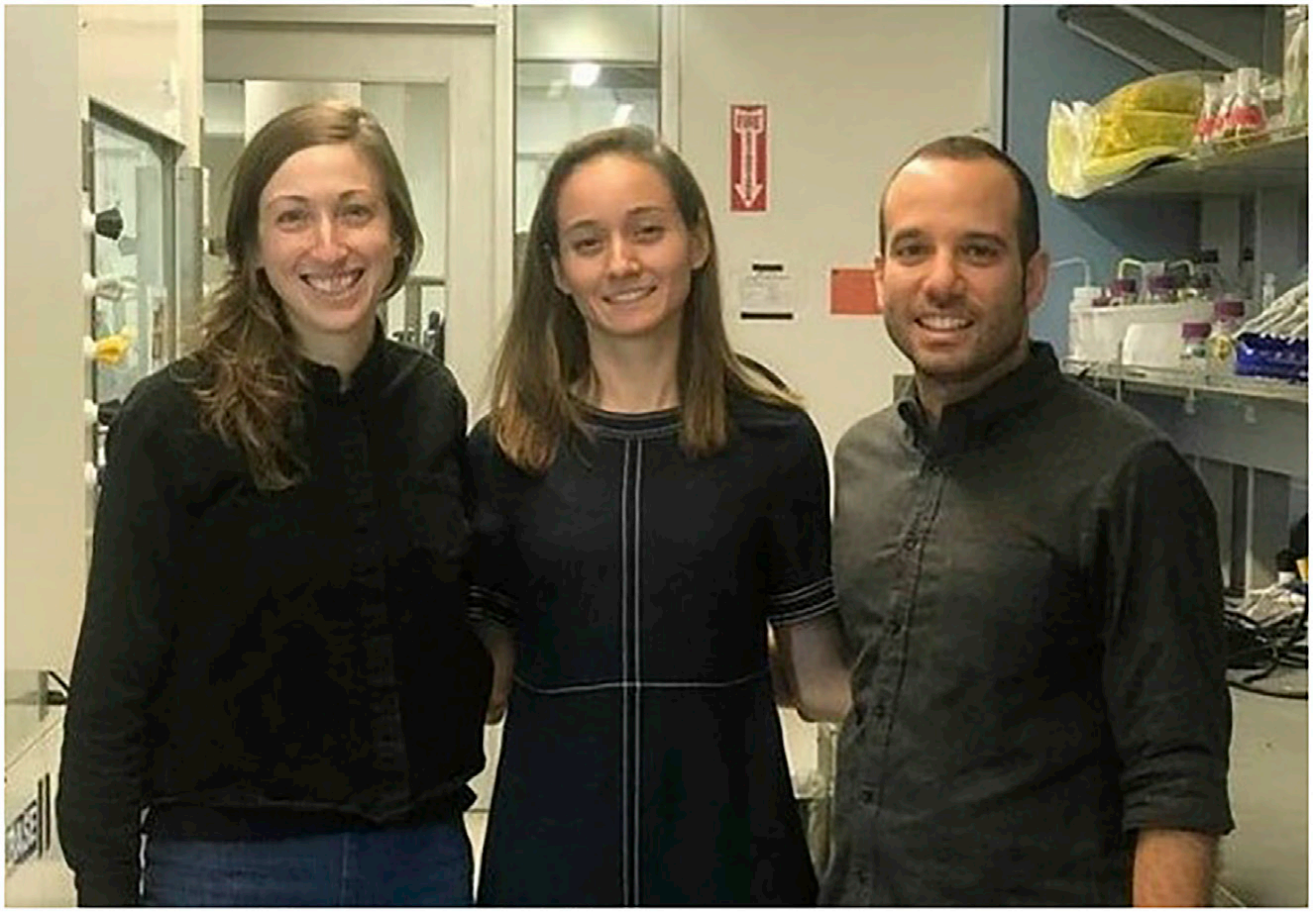

Silverman (left), Nicole Schnable (center), and Modestino (right), after Schnable's master's defense on the development of photocatalytic gels for water treatment. Silverman and Modestino were part of her thesis committee, and the work led to their joint publication.

## Governance: How did the decision of branching out from your fields come about? What implications did it have on your careers (recognition, funding, etc.)?

For scientists, especially early in their careers, it can be perilous to significantly depart from their core disciplines, as it diverts time and resources from advancing their primary research goals. However, the leadership of NYU Tandon and the National Science Foundation, the primary federal sponsor for foundational multi-disciplinary research, have both recognized the importance of supporting faculty who are willing to jump across disciplinary boundaries. For example, NYU offers seed grants to develop robust, technically meritorious, and *convincing* research proposals that are attractive to external sponsors. Among the many challenges that such seed grants help overcome is the development of a common language to describe the research that is accessible for a broad audience and is capable of convincing non-domain experts that are solicited to evaluate the intellectual and real-life impacts of the proposed work.

## Publication: When publishing this or any interdisciplinary paper, how do you decide which community/venue to target? What are the challenges during publication of this or any such research? What initiative (by publishers, funders, etc.) would make communicating interdisciplinary research easier/more effective?

In working together, we became keenly aware that different fields have different expectations about publishing, as well as different timelines needed to produce data or findings. For instance, journals in systems engineering and operations research that accept publications on power grid models and algorithms tend to favor theoretical developments with a simulated application motivated by a larger, real-life challenge. These papers rarely report on specifics of real-life applications and rather envision their future development. On the other hand, there are journals that not only support publications that are well grounded in theory but also validate their results via physical experiments rather than simulations. Naturally, these journals vary not only in the scope of research that they cover but also in the way they process submission (e.g., number of reviewers, expected duration of the review process, tolerance to publishing negative results), which is incredibly important for PhD students, postdocs, and all early career scientists (e.g., tenure-track faculty).

In our experience publishing work at the interface between chemical and environmental engineering, we came to realize that many chemical engineering journals are more focused on material or reactor design, whereas environmental engineering journals are more focused on the use of these materials and reactors to remove or degrade specific environmental contaminants. Given that our collaborative project was a proof-of-concept study for the development of a new material in photocatalytic reactors for water treatment, we decided to publish in the more chemical engineering-focused journal *Reaction Chemistry & Engineering* (https://pubs.rsc.org/en/content/articlelanding/2020/RE/C9RE00456D#!divAbstract). In the future, we plan to submit ongoing work targeted at removal of specific contaminants to journals more closely aligned with environmental engineering research.

Similarly, at the interface of electrical and chemical engineering, we have identified broad energy research journals (e.g., *Joule, Nature Energy, Energy & Environmental Science*) that are receptive to both system-level power grid simulation studies and laboratory experiments typical for chemical engineering. However, although journals may support interdisciplinary research, it has become evident that reviewers from one specific field are not always familiar with the scope of research across different fields and this complicates the evaluation of the potential impact of the work. We anticipate that this situation will change in the future, as more investigators are embracing interdisciplinary research and as the value that it brings starts to become more evident. In addition to peer-reviewed publications, working at the interface of different fields often leads to inventions and thus patents are very important. As an example, our team has worked on grid-integrated electrosynthetic hydrogen generators (US Provisional Patent Application 62/809,429), which serve as building blocks of a larger vision to synergistically integrate water-electricity-chemical networks.

**Figure:**
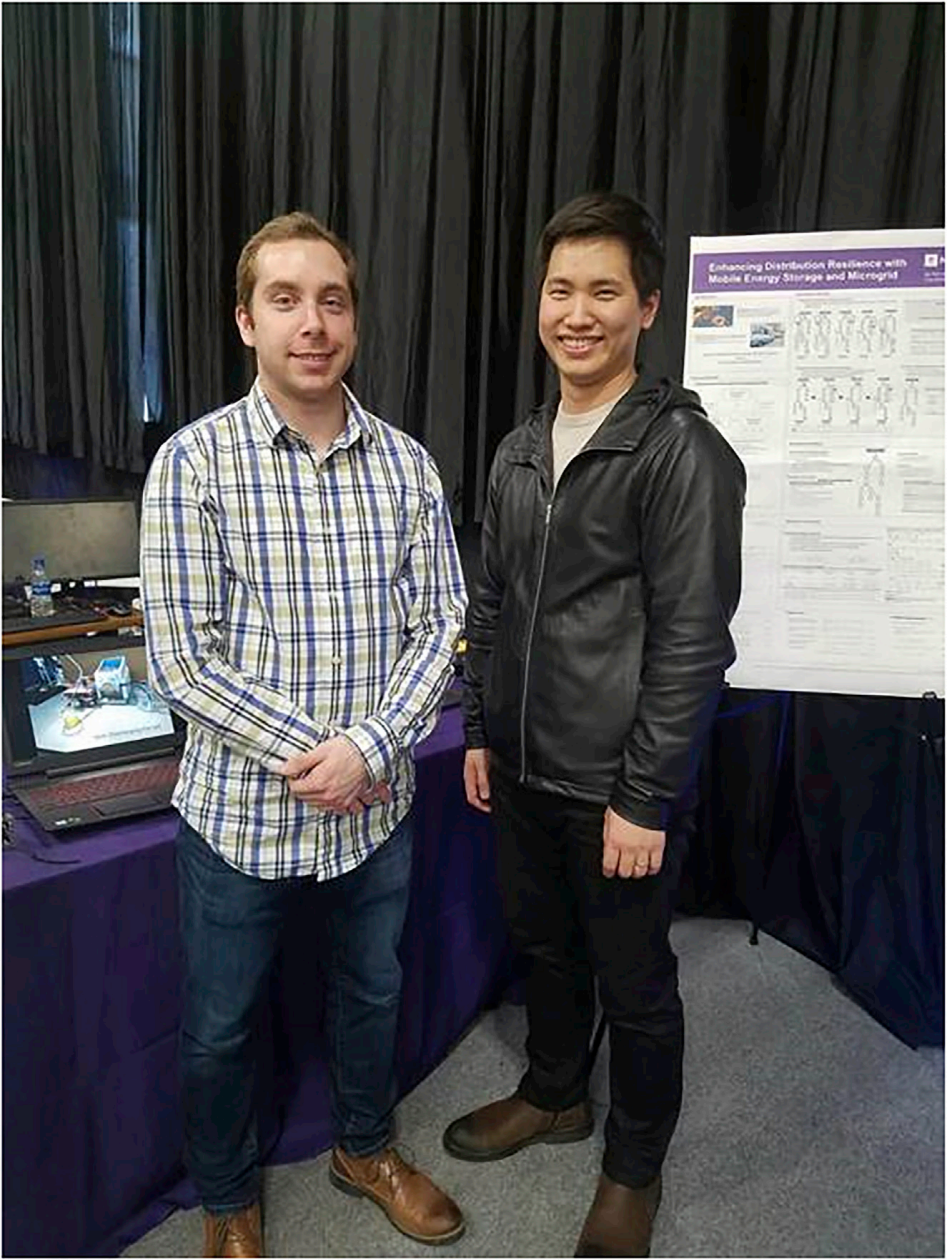

Jip Kim (right), Dvorkin's PhD student, and Daniel Frey (left), Modestino's PhD student, showcased the results of the collaboration between their labs at NYU Tandon's research expo.

## Conclusions/Final Thoughts: What did you learn about interdisciplinary research from the project and what tips would you give to anyone considering undertaking such work? What did your students within this work gain through this interdisciplinary experience?

Finding solutions to complex societal challenges requires interdisciplinary work. We have seen that students trained in interdisciplinary environments develop a broad skillset, have a better understanding of the context and impact of their work, can effectively communicate with colleagues from different fields, and are well prepared to contribute to teams tackling real-world problems in both industry and academia. Interdisciplinary projects involve exploration of new spaces at the interface between different fields, which can inspire students to pursue entrepreneurship opportunities that they identify at this intersection. From our experiences, achieving these benefits is possible if the deep domain-specific knowledge that comes while doing PhD in a given field is not sacrificed but instead augmented with broad interdisciplinary training.

